# The efficacy and safety of ginger supplementation in patients with multiple sclerosis: A rationale and study protocol for a double‐blind randomized controlled trial

**DOI:** 10.1002/hsr2.1004

**Published:** 2022-12-21

**Authors:** Sahar Foshati, Maryam Poursadeghfard, Zahra Heidari, Reza Amani

**Affiliations:** ^1^ Department of Clinical Nutrition, School of Nutrition and Food Science Isfahan University of Medical Sciences Isfahan Iran; ^2^ Clinical Neurology Research Center Shiraz University of Medical Sciences Shiraz Iran; ^3^ Department of Biostatistics and Epidemiology, School of Health Isfahan University of Medical Sciences Isfahan Iran

**Keywords:** ginger, interleukin‐17, matrix metalloproteinase‐9, multiple sclerosis, neurofilament protein L, nitric oxide

## Abstract

**Background and Aims:**

Multiple sclerosis (MS) is a chronic disease characterized by axonal damage, demyelination, inflammation, oxidative stress, and immune cell infiltration. This disease is the first cause of nontraumatic disability in young adults leading to a decline in patients' quality of life. Patients with MS may also suffer from gastrointestinal symptoms due to the disease or prescription drugs. Unfortunately, no treatment for MS has been discovered yet, and prescribed drugs can only help control its clinical course. Interestingly, recent animal studies have shown positive effects of ginger administration in the MS model. Therefore, we aim to determine the effect of ginger supplementation on neurofilament light chain, matrix metalloproteinase‐9, interleukin‐17, nitric oxide, complete and differential blood counts, disability status, quality of life, gastrointestinal symptoms, and body mass index (BMI) in MS patients.

**Methods:**

This study is a double‐blind randomized controlled trial. Fifty‐two patients with relapsing‐remitting MS will be assigned to intervention and control groups using stratified permuted block randomization. The intervention and control groups will take 1500 mg/day ginger and placebo (as corn) supplements for 12 weeks, respectively. All outcomes will be assessed before and after the trial. Serum concentrations of neurofilament light chain, matrix metalloproteinase‐9, and interleukin‐17 will be measured by enzyme‐linked immunosorbent assay. Nitric oxide serum levels will be detected using colorimetry. Complete and differential blood counts will be assessed by an automated hematology analyzer. Disability status, quality of life, and gastrointestinal symptoms will be evaluated by the Expanded Disability Status Scale, MS Impact Scale, and Visual Analog Scale, respectively. BMI will be calculated by dividing weight in kilograms by height in meters squared. Potential side effects of ginger supplementation will also be closely monitored during the study.

**Trial Registration:**

This protocol was registered at the Iranian Registry of Clinical Trials (www.irct.ir) under the registration number IRCT20180818040827N3.

## 1 BACKGROUND

Multiple sclerosis (MS) is a chronic autoimmune, inflammatory, and degenerative disease of the central nervous system (CNS). The etiology of MS is unknown, but some environmental and genetic factors are likely to be contributed to its development. As an example, high body mass index (BMI) can increase the risk of developing MS by about twofold.^1^ Regarding clinical course, this disease is divided into four different subtypes including clinically isolated syndrome, relapsing‐remitting MS, primary progressive MS, and secondary progressive MS. The majority of patients with MS suffer from the relapsing‐remitting type, which is characterized by relapses of MS and periods of stability or remission between relapses.^2^ MS can lead to inflammation, oxidative stress, immune cell infiltration, demyelination, and axonal damage in the human body. Evidence has shown that neurofilament light chain, matrix metalloproteinase‐9, interleukin‐17, nitric oxide, and leukocyte differential count are reliable indicators of axonal damage, demyelination, inflammation, oxidative stress, and infiltration of immune cells in MS patients, respectively.^3^, ^4^ In addition, interleukin‐17 and nitric oxide have been shown to be involved in the pathology and immunomodulation of MS.^5^, ^6^ Unfortunately, no treatment for MS has been discovered so far, and prescription drugs can only help control its clinical course. Therefore, this disease is considered to be the first cause of non‐traumatic disability in young adults leading to a severe reduction in patients' mental and physical quality of life.^7^, ^8^ In clinical practice, the disability status of patients with MS is quantified by the Expanded Disability Status Scale (EDSS).^9^ The guidelines of the European Medicines Agency also suggest EDSS as a primary endpoint in MS trials.^10^ Moreover, gastrointestinal complications are often added to the problems of MS patients. Two‐thirds of them suffer from chronic gastrointestinal symptoms due to the disease or prescribed drugs. The most common symptoms reported by these patients include dysphagia, heartburn, nausea, dyspepsia, diarrhea, constipation, and fecal incontinence.^11^

Ginger (*Zingiber officinale* Roscoe) belongs to the family Zingiberaceae. It has been used as a food, spice, and flavoring for centuries in various societies, especially in Asian countries. It is a therapeutic plant and mainly used to improve gastrointestinal symptoms and relieve pain caused by inflammation in both traditional and modern medicine.^12^, ^13^ Although ginger has been reported to have poor bioavailability due to its low aqueous solubility and excessive phase II hepatic metabolism,^14^ many animal and human studies have shown positive effects after ginger supplementation.^15^ Based on the results of clinical trials, ginger consumption is beneficial for patients with diabetes,^16^ obesity,^17^ and migraine.^18^ It can also improve quality of life and reduce nausea, vomiting, and fatigue induced by chemotherapy in cancer patients.^19^ Moreover, previous studies have shown that ginger can reduce tumor necrosis factor‐alpha and interleukin‐1 beta in patients with knee osteoarthritis.^20^ Furthermore, ginger supplementation can regulate the expression of some inflammatory and immune‐mediating genes such as T‐box transcription factor 21, forkhead box P3, and retinoic acid‐related orphan receptor γt in patients with rheumatoid arthritis and reduce the disease activity score.^21^

Interestingly, recent animal studies have shown that ginger may have positive effects on MS activity. Jafarzadeh et al. (from 2014 to 2017) injected ginger extract at the doses of 200 and 300 mg/kg body weight intraperitoneally into mice induced with experimental autoimmune encephalomyelitis (MS animal model) for 28 days.^22^, ^23^, ^24^, ^25^ Findings from these studies have indicated that ginger supplementation can increase interleukin‐27 and transforming growth factor beta.^22^, ^24^ In addition, it can decrease the clinical symptoms of demyelination, CNS histopathological grades, interferon‐gamma, interleukin‐12, interleukin‐17, interleukin‐23, and interleukin‐33.^22^, ^23^, ^24^ Moreover, ginger administration may reduce the expression of C‐C motif chemokine ligand (CCL) 20 and CCL22 and their receptors, C‐C motif chemokine receptor (CCR) 6 and CCR4, in the spinal cord.^25^ The positive effects of ginger in the MS animal model seem to be exerted through its anti‐inflammatory, antioxidant, neuroprotective, and immunomodulatory properties.^26^

Despite the promising effects of supplementation with ginger in the MS animal model, to the best of our knowledge, no randomized controlled trial (RCT) has been conducted on this topic. Therefore, we aim to determine the impact of ginger supplementation on neurofilament light chain, matrix metalloproteinase‐9, interleukin‐17, nitric oxide, complete and differential blood counts, disability, quality of life, gastrointestinal symptoms, and BMI in patients with MS. The findings of this study will be helpful for people with or at risk of MS, researchers, and clinicians.

## 2 METHODS

### 2.1 Study aims

The main goal of this study is to determine the effect of supplementation with ginger on neurofilament light chain, matrix metalloproteinase‐9, interleukin‐17, nitric oxide, complete and differential blood counts, disability status, quality of life, gastrointestinal symptoms, and BMI in patients with relapsing‐remitting MS. The primary outcomes are interleukin‐17, nitric oxide, and disability status. The secondary outcomes are neurofilament light chain, matrix metalloproteinase‐9, complete and differential blood counts, quality of life, gastrointestinal symptoms, and BMI.

### 2.2 Study design and status

The present study is a parallel double‐blind RCT. It started on 30 Jan. 2022 and is in progress.

### 2.3 Study registration

**Figure 1:**
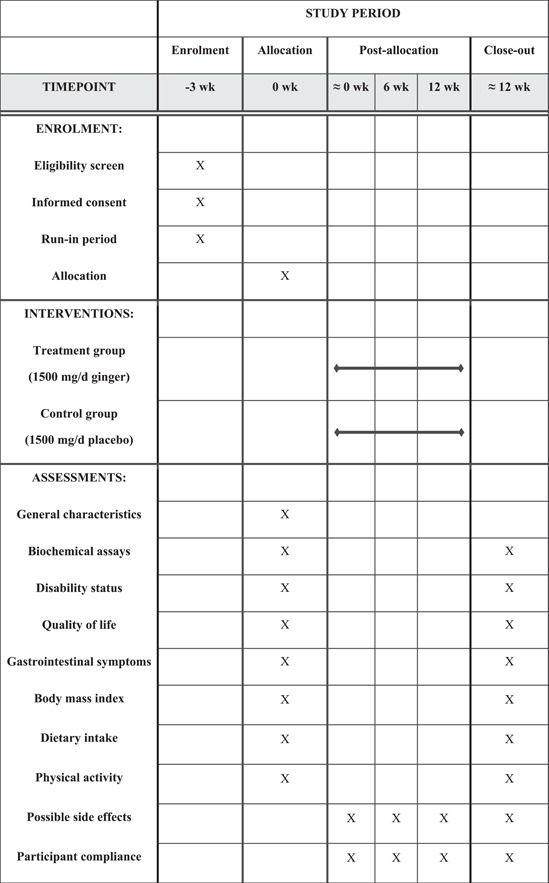
The SPIRIT figure for the schedule of enrollment, interventions, and assessments. SPIRIT, Standard Protocol Items: Recommendations for Interventional Trials.

This trial protocol was registered at www.irct.ir (Iranian Registry of Clinical Trials) on October 06, 2021 under the registration number IRCT20180818040827N3. It was planned and reported based on the Standard Protocol Items: Recommendations for Interventional Trials (SPIRIT) statement.^27^ The schedule of enrollment, interventions, and assessments according to the SPIRIT flow diagram is presented in Figure 1. The populated SPIRIT 2013 checklist is also provided in Supporting Information: Additional File 1.

### 2.4 Study population and setting

The reference population in this trial will be adult patients with relapsing‐remitting MS. The patients will be recruited from Fars MS Association and Imam Reza (A.S) Clinic located in Shiraz, Iran. The prevalence of MS in Iran has been estimated at about 30 cases per 100,000 people.^28^ It has been reported that approximately 70% of Iranian MS patients suffer from the relapsing‐remitting type.^29^

### 2.5 Sample size and sampling method

Given the study design and the existence of two independent groups, the following formula will be used to determine the required sample size:

$$n=\frac{{\left(Z_{1-\frac{\alpha }{2}}+Z_{1-\beta }\right)}^{2}\left(s_{1}^{2}+s_{2}^{2}\right)}{{\left(\mu_{1}-\mu_{2}\right)}^{2}}\;$$

Considering interleukin‐17 as one of the primary outcomes of this study, the number of samples in each of the intervention and control groups was determined to be 13 people (in total 26). The calculations were performed by assuming the type I error equal to 0.05 and the test power equal to 90% and by taking into account *S*_1_ = 4.07, *S*_2_ = 9.19, and the effect size equal to 9.28 based on the previous report.^30^

Considering nitric oxide as one of the primary outcomes of this trial, the number of samples in each of the intervention and control groups was determined to be 22 people (in total 44). The calculations were conducted by assuming the type I error equal to 0.05 and the test power equal to 90% and by taking into account *S*_1_ = 0.72, *S*_2_ = 0.19, and the effect size equal to 0.5 based on the previous report.^31^

Considering disability status measured by EDSS^9^ as one of the primary outcomes of the present study, the number of samples in each of the intervention and control groups was determined to be 22 people (in total 44). The calculations were done by assuming the type I error equal to 0.05 and the test power equal to 90% and by taking into account *S*_1_ = 0.29, *S*_2_ = 1.06, and the effect size equal to 0.74 based on the previous report.^32^

Finally, according to the maximum number of the samples (22 people in each group and 44 in total) and considering a drop‐out rate of ∼20%, the required sample size was estimated to be 26 patients in each group and 52 in total. The participants will be selected using convenience sampling method.

### 2.6 Inclusion and exclusion criteria

The inclusion criteria will include patients with relapsing‐remitting MS according to the 2017 McDonald diagnostic criteria,^33^ age between 18 and 50 years, non‐menopausal women or men, EDSS less than or equal to 4.5,^9^ no MS attack or corticosteroid therapy for at least the last 3 months, no change in the type or dose of MS medications for at least the last 6 months, and the ability and willingness to participate in the study.

The exclusion criteria will include MS patients with other serious diseases (e.g., autoimmune disorders or cancers), pregnancy, MS attack or corticosteroid therapy during the intervention, changes in the type or dose of MS medications during the intervention, supplementation with antioxidants or nutrients (except vitamin D), allergic reactions to ginger or placebo tablets, and consumption of less than 90% of ginger or placebo tablets.

### 2.7 Run‐in period

The participants will be undergone a 3‐week run‐in period before random allocation to the treatment or control group for 12 weeks. During the run‐in period, the patients will be asked to avoid consuming ginger and its products and to maintain their usual diet and physical activity.

### 2.8 Participant allocation

The patients will be assigned to the treatment and control groups using stratified permuted block randomization (block size = 4, allocation ratio = 1:1). The stratification will be done according to gender (female or male). The following valid website will be used for generation of random allocation sequence: https://www.sealedenvelope.com/simple-randomiser/v1/lists. Placebo and ginger tablets will be sealed in sequentially numbered identical bottles based on the allocation sequence. The sequence generation, allocation concealment, and assignment of the patients to the groups will be implemented by different trained persons. The participants, researchers, care providers, and outcome assessors will be blinded to the group assignment.

### 2.9 Intervention

The intervention and control groups will take 500 mg ginger and placebo (corn) tablets three times a day for 12 weeks, respectively. The subjects will be asked to take each of the three tablets along with one of the main meals including breakfast, lunch, and dinner. The dose and duration of ginger supplementation were chosen based on a previous study conducted in patients with active rheumatoid arthritis, which is an autoimmune inflammatory disorder similar to MS.^31^ Appearance characteristics such as packaging, size, shape, and color of placebo tablets will exactly be the same as ginger tablets. Moreover, a very small amount of ginger powder will be poured into each of the boxes containing placebo tablets to make them smell alike, as previously used by Ebrahimzadeh Attari et al.^17^, ^34^ and Mahluji et al.^35^ Both ginger and placebo tablets will be provided by Dineh Iran Industries Complex, Tehran, Iran. Each 500 mg ginger tablet is standardized to contain 25 mg gingerols.

### 2.10 Data collection

The general characteristics of the participants will be examined at the beginning of the study. Biochemical assays, disability status, quality of life, gastrointestinal symptoms, BMI, dietary intake, and physical activity will be evaluated at the beginning and end of the trial. Possible side effects of ginger supplementation will be monitored throughout the study. Laboratory assessments will be performed in Boghrat Pathobiology Laboratory, and nonlaboratory assessments will be done in Imam Reza (A.S) Clinic. Detailed data collection tools are presented below.

#### 2.10.1 General characteristics

The general characteristics of MS patients will be collected through face‐to‐face interviews. These characteristics will include age, gender, marital status, level of education, occupation, duration of MS, number of MS relapses in the previous year, diseases other than MS, type and dose of MS medications, type and dose of other medications used, drug abuse, vitamin D supplementation, smoking habit, and alcohol drinking.

#### 2.10.2 Biochemical assays

After 12 h of fasting, 10 ml of venous blood samples will be taken from the patients. Serum levels of neurofilament light chain, matrix metalloproteinase‐9, and interleukin‐17 will be measured by enzyme‐linked immunosorbent assay using ZellBio GmbH kits. Nitric oxide serum levels will be measured by colorimetric method using KiaZist kit. Complete and differential blood counts will be done using an automated hematology analyzer. Besides, a laboratory specialist will manually double‐check leukocyte differentials by examining blood smears under a microscope.

#### 2.10.3 Disability status

The scores of disability status will be determined through examination by a neurologist (M. P.) using EDSS. EDSS is a ranking scale used to standardize the severity and progression of MS. This scale evaluates the function of seven different systems including the pyramidal tracts, cerebellum, brainstem, tactile nerves, optic nerves, mental function, and enteric and bladder nerves along with the resulting disabilities, particularly in the area of walking. Its scores are in the range of 0−10, and higher scores indicate a greater severity of disability.^9^ It is worth mentioning that the minimal clinically important difference (MCID), the smallest amount of change in an outcome that may be considered important by clinicians or patients, for EDSS has been reported to be between 0.5 and 1.0 points.^36^

#### 2.10.4 Quality of life

The scores of quality of life will be assessed using the self‐report questionnaire of MS Impact Scale (MSIS‐29), which has acceptable validity and reliability in Iranian patients.^37^ This questionnaire contains 29 items, 20 items of which measure the physical impact of MS and 9 items of which measure the psychological impact of MS on patients. Each item has 5 response options with a score of 1−5. Both physical and psychological scales are scored by summing the responses across the relevant items, then converting to a 0−100 scale. The higher the score the worse the quality of life.^38^ It is worth noting that the MCID for MSIS‐29 has been reported to be between 7.0 and 8.0 points.^39^, ^40^

#### 2.10.5 Gastrointestinal symptoms

The frequency and severity of 12 gastrointestinal symptoms including anorexia, dysphagia, heartburn, nausea, vomiting, abdominal pain, bloating, belching, flatulence, constipation, diarrhea, and fecal incontinence will be evaluated using the self‐reported Visual Analog Scale (VAS) ranging from 0 (best) to 100 mm (worst). This method has been previously used by Preziosi et al. to assess gastrointestinal symptoms in MS patients.^41^ The MCID for VAS of gastrointestinal symptoms has been considered 10 mm.^42^

#### 2.10.6 BMI

The participants' weight will be measured to the nearest 0.1 kg in light clothing without shoes using a calibrated digital scale. The participants' height will also be measured to the nearest 0.5 cm in the upright standing position without shoes using a stadiometer. Then, BMI will be calculated by dividing weight in kilograms by height in square meters.

#### 2.10.7 Dietary intake

The usual dietary intake of each patient will be assessed using 3‐day food records. In this method, participants will write down the type and amount of their food intake during 2 weekdays and 1 weekend day. Then, a customized Nutritionist IV software will be used to convert food intake into energy and nutrients.

#### 2.10.8 Physical activity

The usual physical activity of the participants will be assessed using the short form of the International Physical Activity Questionnaire, which has acceptable validity and reliability in Iranians.^43^ This questionnaire consists of 7 items, 6 of which are related to light (walking), moderate, and vigorous physical activity and 1 of which measures total daily sitting time. The level of physical activity will be calculated by multiplying the time allocated to each of the weekly activities by its metabolic equivalent (MET) and then summing the obtained scores. The unit of the total score is MET‐minutes per week. The METs of vigorous, moderate, and light physical activity are 8, 4, and 3.3, respectively.^44^

#### 2.10.9 Possible side effects

Ginger consumption of up to 4000 mg per day is generally recognized as safe by the US Food and Drug Administration.^26^ However, given that the present study is the first RCT on the effect of ginger supplementation in MS patients, possible side effects of ginger or placebo tablets will closely be monitored during the study. This will be done according to the World Health Organization guidelines on safety monitoring of herbal medicines.^45^ Throughout the study, the patients will be encouraged to contact the principal investigator (S. F.) if they suspect any complaint related to the intervention. In addition, the participants will be asked to report side effects and adverse events in weekly phone calls or text messages. Moreover, they will be interviewed for possible side effects (abdominal discomfort, heartburn, diarrhea, etc.) in face‐to‐face visits at the middle and end of the trial.

### 2.11 Participant compliance

The participants will have face‐to‐face visits at the beginning, middle, and end of the study. In addition, adherence to the consumption of tablets will be monitored by weekly phone calls and text messages. Moreover, the subjects will be asked to record the number of consumed tablets by ticking a reminder table. Unused pills will also be taken from patients and counted at the end of the trial.

### 2.12 Potential confounders

At the beginning of the study, multiple potential demographic and medical confounders will be recorded. Moreover, the participants in both groups will be asked to maintain their usual diet and physical activity pattern during the study period and to avoid consuming ginger and other products containing it. To ensure that the participants comply with the protocol, their dietary intake and physical activity will be assessed using the aforementioned tools at the beginning and end of the trial. Finally, all potential confounders will statistically be analyzed and if there is a significant difference between the groups, their effects on study outcomes will be adjusted.

### 2.13 Statistical analysis

SPSS Statistics software version 26 will be used for statistical analysis of data. The Shapiro–Wilk test and skewness will be applied to investigate the normal distribution of quantitative variables. Mean ± standard deviation or median [interquartile range] will be used to report quantitative variables, and number (percentage) will be used to report qualitative variables. For each outcome, the estimated effect size and its 95% confidence interval will be presented. The paired *t*‐test or Wilcoxon signed‐rank test will be performed to compare within‐group differences in quantitative variables. The independent *t*‐test or Mann−Whitney *U* test will be conducted to compare between‐group differences in quantitative variables. The analysis of covariance will be applied to compare between‐group differences by adjusting confounders. The Pearson *χ* ^2^ test or Fisher's exact test will be performed to compare between‐group differences in qualitative variables. All tests will be two‐tailed with a significance level of 0.05. The Statistical Analyses and Methods in the Published Literature (SAMPL) guideline will be followed for reporting statistical results.^46^

## AUTHOR CONTRIBUTIONS

**Sahar Foshati**: Conceptualization; data curation; formal analysis; investigation; methodology; validation; visualization; writing – original draft. **Maryam Poursadeghfard**: Investigation; methodology; validation; writing – review and editing. **Zahra Heidari**: Formal analysis; writing – review and editing. **Reza Amani**: Conceptualization; funding acquisition; methodology; project administration; supervision; writing – review and editing.

## CONFLICT OF INTEREST

The authors declare no conflict of interest.

## ETHICS STATEMENT

This study is in agreement with the Declaration of Helsinki and its later amendments and has already been approved by the Medical Ethics Committee of Isfahan University of Medical Sciences with the ethics code IR.MUI.RESEARCH.REC.1400.248. Before enrollment, both oral and written informed consent will be obtained from the participants.

## TRANSPARENCY STATEMENT

The lead author Reza Amani affirms that this manuscript is an honest, accurate, and transparent account of the study being reported; that no important aspects of the study have been omitted; and that any discrepancies from the study as planned (and, if relevant, registered) have been explained.

## Supporting information

Supporting information.

## Data Availability

Data sharing is not applicable to this article because no datasets are currently generated or analyzed.
